# Clinical outcomes of metastatic non-clear cell renal cell carcinoma: a real-world single-centre experience

**DOI:** 10.1080/07853890.2026.2613590

**Published:** 2026-01-23

**Authors:** Jie Wu, Chuan-Zhen Cao, Hong-Lei Cui, Man-Xiang Chen, Li-Na Gao, Shan Zheng, Ai-Ping Zhou, You-Yan Guan, Xin-Gang Bi, Hong-Zhe Shi, Jian-Zhong Shou

**Affiliations:** a Department of Urology, Zhejiang Cancer Hospital, Hangzhou, P. R. China; b Department of Urology, National Cancer Center/National Clinical Research Center for Cancer/Cancer Hospital, Chinese Academy of Medical Sciences and Peking Union Medical College, Beijing, P. R. China; c Hangzhou Institute of Medicine (HIM), Chinese Academy of Sciences, Hangzhou, P. R. China; d Department of Urology, China-Japan Friendship Hospital, Beijing, P. R. China; e Department of Pathology, National Cancer Center/National Clinical Research Center for Cancer/Cancer Hospital, Chinese Academy of Medical Sciences and Peking Union Medical College, Beijing, P. R. China; f Department of Medical Oncology, National Cancer Center/National Clinical Research Center for Cancer/Cancer Hospital, Chinese Academy of Medical Sciences and Peking Union Medical College, Beijing, P. R. China

**Keywords:** Non-clear cell renal cell carcinoma, pathology, metastasis, systemic treatment, prognosis

## Abstract

**Purpose:**

In the current study, we aimed to characterize the clinicopathological features of metastatic non-clear cell renal cell carcinoma (nccRCC) using retrospective data from our centre and to assess the clinical outcomes of patients treated with first-line immunotherapy-tyrosine kinase inhibitor (IO-TKI) therapy versus TKI monotherapy.

**Methods:**

We conducted retrospective analysis of 105 metastatic nccRCC patients at our centre from Jan 2006 to Oct 2022. The end points included progression-free survival (PFS) and overall survival (OS). Survival analysis was performed using the Kaplan–Meier curve and multivariate Cox regression model.

**Results:**

Among metastatic nccRCC patients, the most prevalent histology was papillary RCC (39.0%). The most common distant metastasis site was lung metastasis (52.4%), followed by bone metastasis (33.3%). The proportion of the favourable, intermediate and poor IMDC risk group were 19.0%, 54.3% and 26.7%, respectively. Among metastatic nccRCC patients, 26 received first-line IO-TKI therapy and 79 received first-line TKI monotherapy therapy. During the median follow-up of 22.2 months, the 1-year PFS and 3-year OS of IO-TKI group was 46.2% and 48.0%, respectively. In comparison, the 1-year PFS and 3-year OS of TKI monotherapy group was 34.9% and 33.7%, respectively. According to the Cox regression model, unclassified RCC pathology (HR = 2.027, *p* = 0.023) and poor IMDC risk group (HR = 2.285, *p* = 0.011) were identified as the independent risk factors for PFS. Poor IMDC risk group (HR = 2.638, *p* = 0.007) was also identified as the independent risk factors for OS.

**Conclusion:**

For metastatic nccRCC, unclassified RCC pathology and poor IMDC risk group were identified as independent risk factors for poor prognosis and IO-TKI showed promising efficacy in patients with metastatic nccRCC.

## 1. Introduction

Historically, renal cell carcinoma is generally subdivided into two principal subtypes: clear cell renal cell carcinoma (ccRCC) and non-clear cell renal cell carcinoma (nccRCC), accounting for approximately 80% and 20% of all RCC cases, respectively [1]. nccRCC is a clinicopathological heterogeneous disease that comprises a complex mixture of different pathology subtypes, including papillary RCC (pRCC), chromophobe RCC (chRCC), unclassified RCC and other entities according to the World Health Organization (WHO) classification [2,3]. Metastatic non-clear cell renal cell carcinoma (metastatic nccRCC) is the end stage of this disease, with a poor prognosis. According to survival data from the International mRCC Database Consortium (IMDC), patients with metastatic mpRCC experience markedly poorer outcomes compared with mccRCC, with a median OS of 11.0–15.6 months for mpRCC versus 17.6–25.1 months for mccRCC [4].

Due to its heterogeneity and rarity, most phase 3 clinical trials investigating treatments for mRCC excluded nccRCC patients [5]. Consequently, the absence of level 1 evidence in clinical guidelines has contributed to poorly defined standard treatment for metastatic nccRCC. Based on the data from some phase II clinical trials and meta-analyses, the immunotherapy-tyrosine kinase inhibitor (IO-TKI) therapy or TKI monotherapy has shown certain efficacy for metastatic nccRCC patients. However, these agents were primarily developed based on the design of ccRCC trials, and the objective response rate (ORR) in metastatic nccRCC remains relatively lower compared to metastatic ccRCC [6–9]. Therefore, enrolment in clinical trials is recommended by the NCCN guidelines and the updated EAU guidelines for metastatic nccRCC patients [10].

In the current study, we aimed to: (1) characterize the clinicopathological features of metastatic nccRCC based on a 20-year retrospective cohort at our centre; and (2) evaluate the clinical outcomes of patients treated with first-line IO-TKI therapy versus TKI monotherapy.

## 2. Method

### 2.1. Patients’ selection strategy

We conducted retrospective analysis of metastatic nccRCC patients at our centre from Jan 2006 to Oct 2022. Only patients who met the following criteria were included: (1) pathological confirmed as nccRCC; (2) fluorescence *in situ* hybridization (FISH) or genetic testing were available for genetically defined subtypes, such as TFE3/TFEB-rearranged RCC and fumarate hydratase-deficient RCC (FH-RCC); (3) presented distant metastasis and received at least one line of the TKI monotherapy or IO-TKI therapy; and (4) adequate tumour data and follow-up information were available. Demographic, clinicopathological and systemic therapy data were collected. All available pathology slides were re-reviewed by two experienced urological pathologists (*Li-Na Gao* and *Shan Zheng*) according to the fifth edition of the WHO Classification of Urinary System Tumours. The primary end point was progression-free survival (PFS), defined as the time from initiation of systemic treatment to the first documented disease progression. The secondary end point was OS, defined as the time from initiation of treatment to death from any cause or last follow-up. CT scans were performed every 4–8 weeks after initiation of systemic treatment and tumour response was assessed according to the Response Evaluation Criteria in Solid Tumors (RECIST) version 1.1. This study was approved by the institutional ethics committee of National Cancer Center/National Clinical Research Center for Cancer/Cancer Hospital, Chinese Academy of Medical Sciences and Peking Union Medical College (20/245-2441) and conducted in accordance with the Declaration of Helsinki. Written informed consent was obtained from all the patients.

### 2.2. Statistical analysis

The statistical analysis was performed base on the R software version 4.2.2 (http://www.R-project.org). Survival analysis was performed using the Kaplan–Meier curve and multivariate Cox regression model based on the ‘survminer’ and ‘survival’ packages. The forest plot was created using the ‘forestplot’ package, and the Venn diagram was generated using the ‘ggvenn’ package. All statistical analysis was evaluated at a two-sided *p* value of 0.05.

## 3. Result

### 3.1. Clinicopathologic characteristics of metastatic nccRCC patients

Overall, 105 metastatic nccRCC patients were enrolled, with a median follow-up of 22.2 months. The most prevalent histology was papillary RCC (41, 39.0%). Notably, the second most common histological type was TFE3/TFEB-rearranged RCC (31, 29.5%), followed by unclassified RCC (26, 24.8%). Other histology types included chromophobe RCC (3), FH-RCC (3) and collecting duct carcinoma (1). The median age of metastatic nccRCC patients was 50 years old and the majority of patients were male (69.5%). 68.6% patients had advanced primary tumour stage of T3/4 and 48.6% patients had regional lymph node metastasis. The most common distant metastasis site was lung metastasis (52.4%), followed by bone metastasis (33.3%) and 21.0% patients had liver metastasis (Supplementary Figure 1). The proportion of the favourable, intermediate and poor IMDC risk group were 19.0%, 54.3% and 26.7%, respectively. Among metastatic nccRCC patients, 26 received first-line IO-TKI therapy, treatment regimen was axitinib plus pembrolizumab; 79 patients first-line TKI monotherapy therapy treatment regimens included sunitinib (*n* = 42), sorafenib (*n* = 21) and pazopanib (*n* = 16) (Table 1). As of data cutoff (Oct, 2023), 98 patients (93.3%) experienced tumour progression and 74 patients (70.5%) had died.

**Table 1.** Demographic and clinical characteristics of metastatic non-clear cell renal cell carcinoma patients.

Variable		TKI (*n* = 79)	IO-TKI (*n* = 26)	*p* value
Age at diagnosis	Mean (SD)	48.87 (16.11)	50.15 (16.56)	0.730
Gender	Male	57 (72.2)	16 (61.5)	0.439
	Female	22 (27.8)	10 (38.5)	
Pathology	Papillary	27 (34.2)	14 (53.8)	**0.037**
	TFE3/TFEB	29 (36.7)	2 (7.7)	
	Unclassified	18 (22.8)	8 (30.8)	
	Chromophobe	2 (2.5)	1 (3.8)	
	FH-deficient	2 (2.5)	1 (3.8)	
	Collecting duct	1 (1.3)	0 (0.0)	
T stagedf	T1	16 (20.3)	3 (11.5)	0.434
	T2	11 (13.9)	3 (11.5)	
	T3	43 (54.4)	19 (73.1)	
	T4	9 (11.4)	1 (3.8)	
N stage	N0	36 (45.6)	15 (57.7)	0.397
	N1	43 (54.4)	11 (42.3)	
Metastatic site	Bone			0.936
	Yes	27 (34.2)	8 (30.8)	
	No	52 (65.8)	18 (69.2)	
	Lung			0.158
	Yes	45 (57.0)	10 (38.5)	
	No	34 (43.0)	16 (61.5)	
	**Liver**			**0.024**
	Yes	12 (15.2)	10 (38.5)	
	No	67 (84.8)	16 (61.5)	
	Other			0.523
	Yes	35 (44.3)	9 (34.6)	
	No	44 (55.7)	17 (65.4)	
Metastatic time	Synchronous	51 (64.6)	17 (65.4)	1.000
	Metachronous	28 (35.4)	9 (34.6)	
IMDC risk groups	Favourable	14 (17.7)	6 (23.1)	0.787
	Intermediate	43 (54.4)	14 (53.8)	
	Poor	22 (27.8)	6 (23.1)	
Surgery	Nephrectomy	68 (86.1)	17 (65.4)	**0.040**
	No surgery	11 (13.9)	9 (34.6)	

IMDC, the International mRCC Database Consortium; TKI, tyrosine kinase inhibitor; IO-TKI, the immunotherapy-tyrosine kinase inhibitor. Bold values indicate *p* < 0.05..

Given the long study period of this single-centre retrospective analysis, we compared baseline characteristics between patients treated during 2006–2018 and 2019–2022 (Supplementary Table 1). The primary difference was the shift from TKI monotherapy towards IO–TKI combinations in recent years, while the temporal changes in treatment patterns did not substantially alter baseline disease features.

### 3.2. The efficacy of IO-TKI and TKI monotherapy for metastatic nccRCC patients

**Figure 1.**
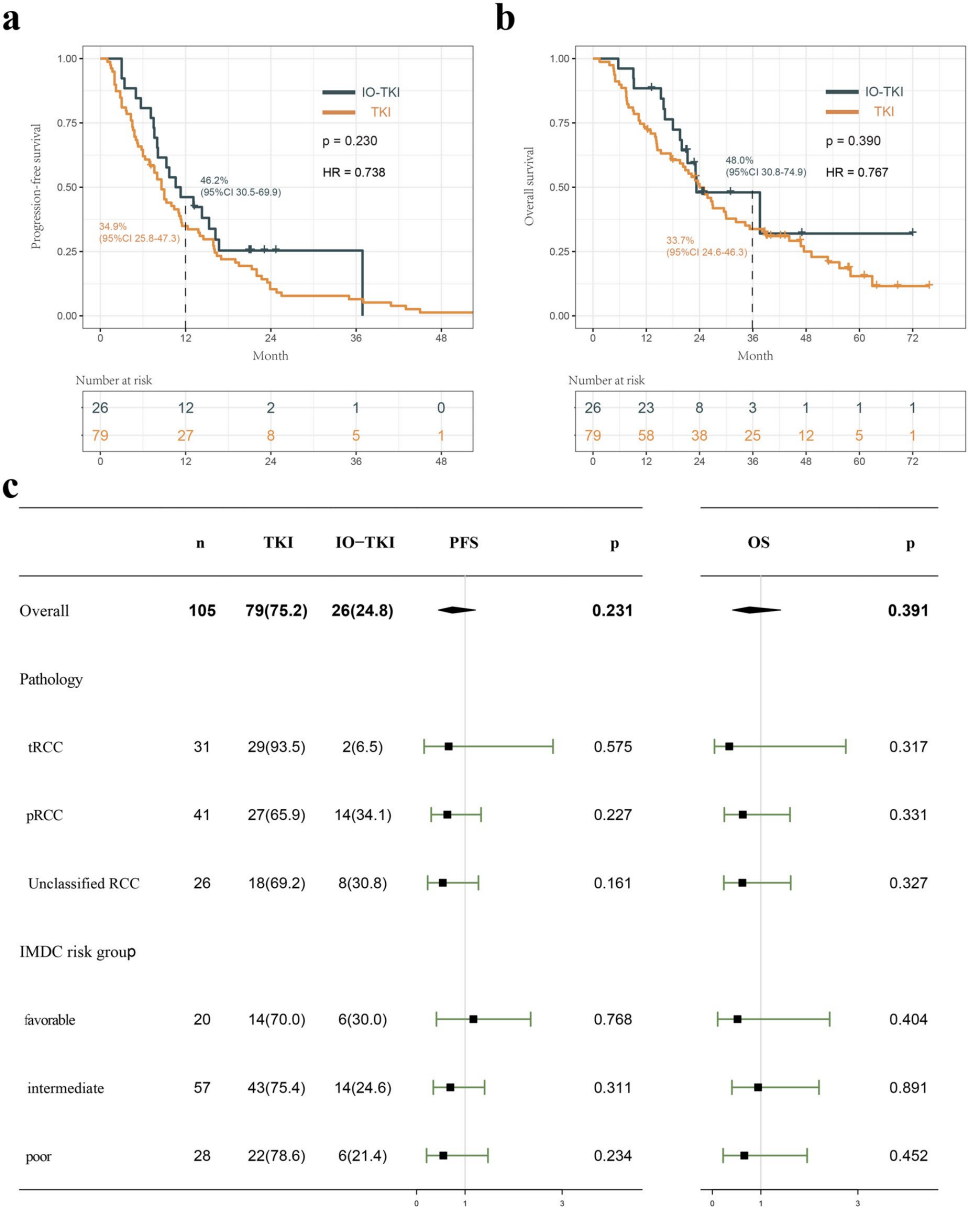
Kaplan-Meier curves for PFS (a) and OS (b) of metastatic nccRCC patients according to first-line treatment strategy. (c) Forest plots summarizing the HRs of PFS and OS in metastatic nccRCC patients stratified by first-line treatment strategy.

The 1-year PFS and 3-year OS of IO-TKI group was 46.2% and 48.0%, respectively. In comparison, the 1-year PFS and 3-year OS of TKI monotherapy group was 34.9% and 33.7%, respectively (Figure 1a and 1b). In addition, subgroup analyses were performed to further compare the efficacy of IO-TKI and TKI monotherapy for metastatic nccRCC patients among groups based on pathology and IMDC risk group (Figure 1c). The results suggest that IO-TKI may offer superior efficacy compared to TKI monotherapy for patients with metastatic nccRCC. However, the differences were not statistically significant.

### 3.3. Prognostic factor for metastatic nccRCC

**Figure 2.**
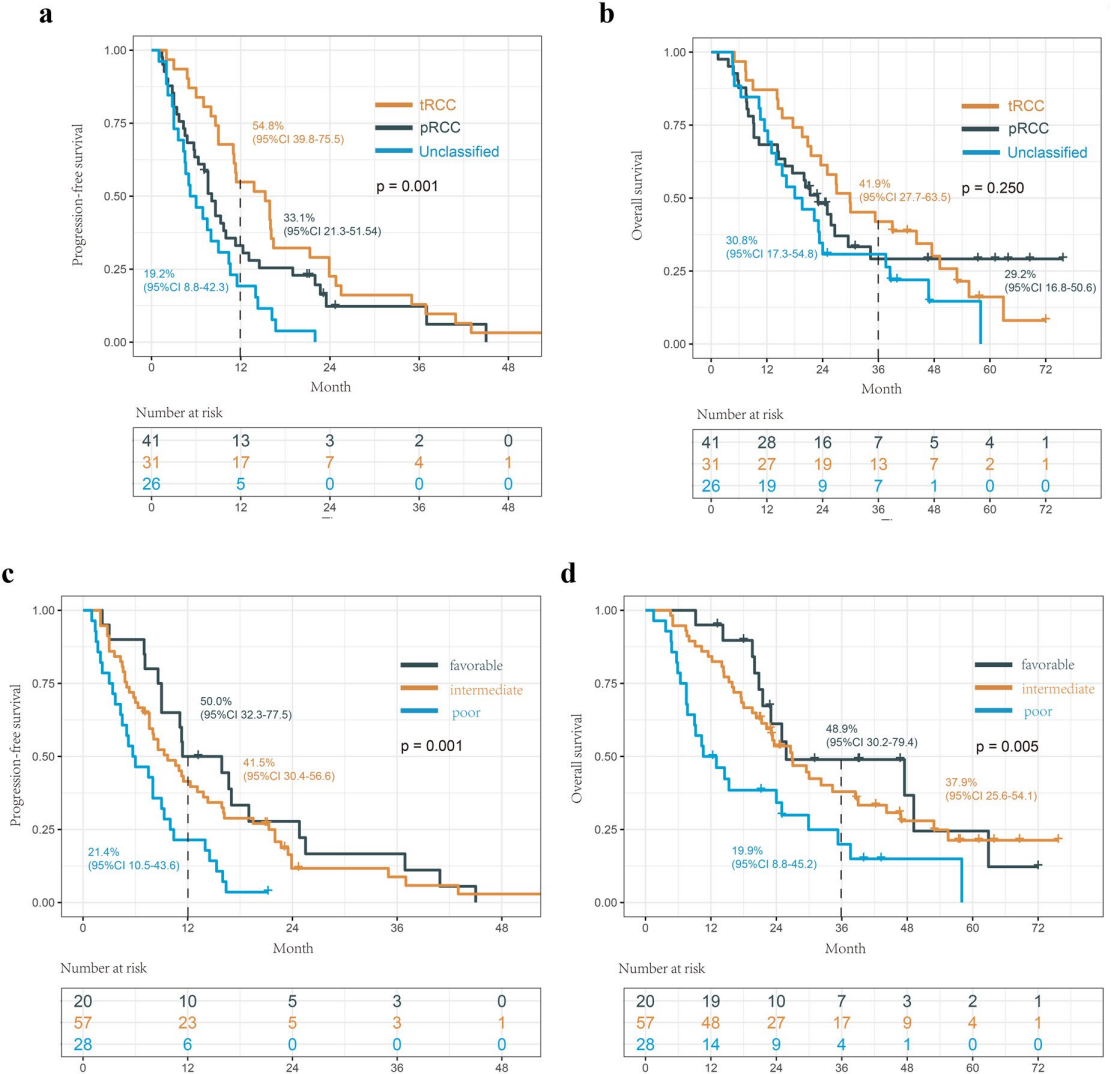
Kaplan-Meier curves for PFS (a) and OS (b) of metastatic nccRCC patients stratified by pathological type. Kaplan-Meier curves for PFS (c) and OS (d) of metastatic nccRCC patients stratified by IMDC risk group.

According to the Cox regression model, unclassified RCC pathology (HR = 2.027, *p* = 0.023) and poor IMDC risk group (HR = 2.285, *p* = 0.011) were identified as the independent risk factors for PFS (Supplementary Table 2). Poor IMDC risk group was also identified as the independent risk factors for OS (HR = 2.638, *p* = 0.007) (Supplementary Table 3). Notably, the PFS of TFE3/TFEB-rearranged RCC was significantly longer than papillary RCC and unclassified RCC (*p* = 0.001), with a median PFS of 15.3, 8.1 and 6.0 months, respectively (Figure 2a). The OS of tRCC was also longer than pRCC and unclassified RCC, with a median OS of 29.9, 23.0 and 18.8 months, respectively, while the difference was not significant (*p* = 0.250) (Figure 2b). Notably, although the prognosis of the poor IMDC risk group was the worst, there was no statistical significance between the survival of the favourable and the intermediate IMDC risk group (Figure 2c and 2d). The clinical outcomes of patients with rare metastatic nccRCC pathology subtypes were further presented in Supplementary Figure 2.

## 4. Discussion

NccRCC are histopathologically heterogeneous diseases that contain various histological subtypes. With deeper understanding of these diseases, emerging entities are being identified, each characterized by distinct morphological and molecular features [11]. In the 2016 WHO classification of renal tumours, several novel molecularly defined nccRCC were introduced, including MiT family translocation RCC and FH-RCC [12]. More molecularly driven nccRCC classifications has been further recognized in the 2022 WHO classification, including ELOC (TCEB1)-mutated RCC, ALK-rearranged RCC and SMARCB1-deficient medullary RCC [2,3]. Additionally, MiT family translocation RCC was further subdivided into TFE3-rearranged RCC and TFEB-altered RCC subtypes [13]. Notably, the 2022 WHO classification introduced significant change to pRCC, discarding the morphology-based classification of the type 1/2 pRCC. Recent molecular studies suggest that the type 2 pRCC may not a single well-defined category, but rather a mix of entities with different tumour phenotypes and divergent molecular backgrounds. These new tumour entities are now classified as independent RCC pathological subtypes, such as FH-RCC [14]. However, although there has been substantial evolution in the pathological and molecular understanding of nccRCC over the past decade, research and evidence regarding its treatment remain limited.

The treatment paradigm of metastatic ccRCC has evolved from cytokine regimens to TKI targeted therapy over the past two decades. The randomized phase 3 trials KEYNOTE-426, CheckMate 9ER and CLEAR have further demonstrated the efficiency of immunotherapy-based drug combinations as first-line treatment for metastatic ccRCC [15–17]. However, due to the heterogeneity and rarity of nccRCC, each with distinct biological and clinical features, the treatment strategy and clinical outcome varies according to pathological type. While most clinical trials generally combined nccRCC cases into a single cohort, which primarily consisted of pRCC, leading to the insufficient data for other nccRCC rare types [18]. Currently, treatment strategy for metastatic nccRCC has not been optimized. IO-TKI or TKI monotherapy has shown certain efficacy for metastatic nccRCC patients according to some phase II clinical trials. The results of ESPN and ASPEN trials revealed that the PFS for metastatic nccRCC patients received sunitinib was 6.1 and 8.3 months, respectively [6,7]. Cabozantinib, as a novel multiple TKI targeting MET and VEGFR, has been demonstrated efficacy for metastatic nccRCC treatment, with the ORR of 27% and the PFS of 6.9 months [19]. In 2021, the results of SWOG 1500 trial further showed encouraging activity of cabozantinib for metastatic nccRCC, with the ORR of 23% and the PFS of 9 months, significant better than patients in sunitinib cohort [20].

Recent trials have also demonstrated the efficacy of immune checkpoint inhibitors (ICIs) in metastatic nccRCC. KEYNOTE-427 is the first phase II study investigating the efficacy and safety of ICIs (pembrolizumab monotherapy) as first-line treatment for patients with metastatic nccRCC. The study enrolled 165 patients, the median PFS and OS was 4.2 and 28.9 months, respectively. The ORR of pRCC, chRCC and unclassified RCC was 28.8%, 9.5% and 30.8%, respectively [21]. Another phase II trial conducted by MSKCC confirmed promising efficacy of cabozantinib plus nivolumab in metastatic nccRCC. The ORR of the Cohort 1 (32 pRCC, 6 unclassified RCC and 2 tRCC) was 47.5%, with the median PFS and OS of 12.5 months and 28 months, respectively. While patients in the Cohort 2 (7 chRCC) show no responses to the treatment, with the ORR of 0% [8,22]. KEYNOTE-B61 is a multi-centre phase 2 trial assessing the efficacy of Lenvatinib plus pembrolizumab for patients with advanced nccRCC. The results revealed durable antitumour activity of the treatment, with the ORR and median PFS of 49% and 18 months, respectively [23]. The SUNNIFORECAST trial is a randomized phase II study evaluating ipilimumab plus nivolumab versus standard of care (TKI or IO–TKI) in patients with nccRCC. The 12-month OS and median OS in the ipilimumab/nivolumab arm were 78% and 33.2 months, respectively, demonstrating a promising clinical benefit for dual immunotherapy in this heterogeneous population [9]. In the current study, 26 nccRCC patients received IO-TKI therapy. The median PFS and OS in this group were 10.9 and 23.3 months, respectively. These real-world results further support the promising efficacy of IO-TKI combinations for metastatic nccRCC.

In conclusion, this study retrospectively analyzed the clinicopathological and prognostic data of 105 metastatic nccRCC patients at our centre, including 41 pRCC, 31 TFE3/TFEB-rearranged RCC, 26 unclassified RCC, 3 chRCC, 3 FH-RCC and 1 collecting duct carcinoma. Unclassified RCC pathology and poor IMDC risk group were the independent risk factors for poor prognosis. Our results suggested that IO-TKI may offer superior efficacy compared to TKI monotherapy for patients with metastatic nccRCC. However, due to the limitations of the sample size, the differences were not statistically significant.

Some limitations of our study must be noted. First, the study is a single-centre retrospective analysis conducted over a considerable time span, which may introduce a risk of selection bias. Second, our results are limited by the relatively small patient cohort, especially for rare nccRCC pathology subtypes. In addition, comprehensive genomic information was not available because many archived tumour specimens did not meet the quality requirements for contemporary RNA sequencing. Despite the limitations of this study, our results provided real-world data regarding the clinical and prognostic characteristics of metastatic nccRCC patients.

## Supplementary Material

Supplemental Material

Supplemental Material

Supplemental Material

## Data Availability

All data generated or analyzed during this study are included in this published article.
