# Supplementary material for: Clinical outcomes of metastatic non-clear cell renal cell carcinoma: a real-world single-centre experience
Source: Ann Med. 2026 Jan 23;58(1):2613590. doi: 10.1080/07853890.2026.2613590 (PMC12833892; doi:10.1080/07853890.2026.2613590)
Supplement: Supplemental Material [file IANN_A_2613590_SM2587.docx]

**Supplementary Figure 1** Venn diagram illustrating the distant metastatic sites of metastatic nccRCC.

**Supplementary Figure 2** Swimmer plot showing the survival of patients with rare metastatic nccRCC pathology subtypes. This cohort contained three FH-RCC, three chRCC, two TFEB-rearranged RCC, and one collecting duct RCC. FH-RCC1 was a 32-year-old female with initial stage of T2N0M0. 17.4 months after nephrectomy, FH-RCC1 developed liver and bone metastasis. FH-RCC1 then received first-line axitinib plus pembrolizumab and was alive at 13.2-month follow-up. FH-RCC2 was a 26-year-old male with lung, bone and lymph node metastasis at initial diagnosis (T4N1M1). FH-RCC2 received first-line sunitinib monotherapy and experienced tumor progression at 8-month follow-up. FH-RCC2 then received second-line axitinib plus pembrolizumab and was alive at 35.2-month follow-up. FH-RCC3 was a 25-year-old male with bone and lymph node metastasis at initial diagnosis (T3N1M1). FH-RCC3 received first-line sunitinib monotherapy and experienced tumor progression at 19.5-month follow-up.

**Supplementary Table 1. Comparison of clinical characteristics of metastatic non–clear cell renal cell carcinoma patients diagnosed during 2006–2018 versus 2019–2022**

| Variable |  | 2005–2018  (n=54) | 2019–2022  (n=51) | *p* value |
| --- | --- | --- | --- | --- |
| T stage | T1 | 11 (20.4) | 8 (15.7) | 0.746 |
|  | T2 | 8 (14.8) | 6 (11.8) |  |
|  | T3 | 29 (53.7) | 33 (64.7) |  |
|  | T4 | 6 (11.1) | 4 (7.8) |  |
| N stage | N0 | 25 (46.3) | 26 (51.0) | 0.776 |
|  | N1 | 29 (53.7) | 25 (49.0) |  |
| Metastatic site | Bone |  |  | 0.300 |
|  | Yes | 21 (38.9) | 14 (27.5) |  |
|  | No | 33 (61.1) | 37 (72.5) |  |
|  | Lung |  |  | 0.635 |
|  | Yes | 30 (55.6) | 25 (49.0) |  |
|  | No | 24 (44.4) | 26 (51.0) |  |
|  | Liver |  |  | 0.178 |
|  | Yes | 8 (14.8) | 14 (27.5) |  |
|  | No | 46 (85.2) | 37 (72.5) |  |
|  | Other |  |  | 0.216 |
|  | Yes | 19 (35.2) | 25 (49.0) |  |
|  | No | 35 (64.8) | 26 (51.0) |  |
| Metastatic time | Synchronous | 35 (64.8) | 33 (64.7) | 1.000 |
|  | Metachronous | 19 (35.4) | 18 (35.3) |  |
| IMDC risk groups | Favorable | 8 (14.8) | 12 (23.5) | 0.320 |
|  | Intermediate | 33 (61.1) | 24 (47.1) |  |
|  | Poor | 13 (24.1) | 15 (29.4) |  |
| **First-line treatment** | TKI | 54 (100.0) | 25 (49.0) | **< 0.001** |
|  | IO-TKI | 0 (0.0) | 26 (51.0) |  |
| Surgery | Nephrectomy | 47 (87.0) | 38 (74.5) | 0.166 |
|  | No surgery | 7 (13.0) | 13 (25.5) |  |

IMDC, the International mRCC Database Consortium; TKI, tyrosine kinase inhibitor; IO-TKI, the immunotherapy-tyrosine kinase inhibitor.

**Supplementary Table** **2 Univariate and multivariate Cox regression analysis of progression-free survival in metastatic non-clear cell renal cell carcinoma patients**

|  | Univariate analysis | | | Multivariate analysis | | |
| --- | --- | --- | --- | --- | --- | --- |
|  | HR | 95%CI | *p* value | HR | 95%CI | *p* value |
| Age |  |  |  |  |  |  |
| ≤70 | Ref |  |  |  |  |  |
| >70 | 0.928 | 0.462-1.864 | 0.833 |  |  |  |
| Gender |  |  |  |  |  |  |
| Male | Ref |  |  | Ref |  |  |
| Female | 0.676 | 0.431-1.061 | 0.088 | 0.831 | 0.517-1.335 | 0.444 |
| **Pathology** |  |  |  |  |  |  |
| TFE3/TFEB | Ref |  |  | Ref |  |  |
| Papillary | 1.517 | 0.928-2.479 | 0.096 | 1.516 | 0.924-2.488 | 0.100 |
| Unclassified | 2.853 | 1.607-5.064 | **＜0.001** | 1.999 | 1.067-3.748 | **0.031** |
| Other | 1.304 | 0.587-2.893 | 0.514 | 1.123 | 0.491-2.565 | 0.784 |
| T stage |  |  |  |  |  |  |
| T1-2 | Ref |  |  |  |  |  |
| T3-4 | 1.073 | 0.702-1.642 | 0.744 |  |  |  |
| N stage |  |  |  |  |  |  |
| N0 | Ref |  |  |  |  |  |
| N1 | 1.104 | 0.736-1.655 | 0.633 |  |  |  |
| **Metastatic site** |  |  |  |  |  |  |
| ***Bone*** |  |  |  |  |  |  |
| No | Ref |  |  | Ref |  |  |
| Yes | 1.585 | 1.023-2.454 | **0.039** | 1.441 | 0.884-2.350 | 0.143 |
| *Lung* |  |  |  |  |  |  |
| No | Ref |  |  |  |  |  |
| Yes | 0.939 | 0.628-1.404 | 0.760 |  |  |  |
| *Liver* |  |  |  |  |  |  |
| No | Ref |  |  |  |  |  |
| Yes | 0.799 | 0.478-1.336 | 0.392 |  |  |  |
| Metastatic time |  |  |  |  |  |  |
| Synchronous | Ref |  |  |  |  |  |
| Metachronous | 1.102 | 0.728-1.666 | 0.647 |  |  |  |
| **IMDC risk groups** |  |  |  |  |  |  |
| Favorable | Ref |  |  | Ref |  |  |
| Intermediate | 1.302 | 0.765-2.216 | 0.330 | 1.071 | 0.612-1.876 | 0.810 |
| Poor | 2.747 | 1.484-5.087 | **0.001** | 2.162 | 1.121-4.168 | **0.021** |
| Therapy |  |  |  |  |  |  |
| TKI | Ref |  |  |  |  |  |
| IO-TKI | 0.738 | 0.449-1.213 | 0.231 |  |  |  |

HR, hazard ratio; CI, confidence interval; IMDC, the International mRCC Database Consortium; TKI, tyrosine kinase inhibitor; IO-TKI, the immunotherapy-tyrosine kinase inhibitor.

The bold values indicated that p < 0.05.

**Supplementary Table 3 Univariate and multivariate Cox regression analysis of overall survival in metastatic non-clear cell renal cell carcinoma patients**

|  | Univariate analysis | | |
| --- | --- | --- | --- |
|  | HR | 95%CI | *p* value |
| Age |  |  |  |
| ≤70 | Ref |  |  |
| >70 | 0.857 | 0.371-1.978 | 0.717 |
| Gender |  |  |  |
| Male | Ref |  |  |
| Female | 1.174 | 0.721-1.912 | 0.519 |
| Pathology |  |  |  |
| TFE3/TFEB | Ref |  |  |
| Papillary | 1.111 | 0.639-1.933 | 0.709 |
| Unclassified | 1.641 | 0.906-2.975 | 0.103 |
| Other | 0.562 | 0.169-1.873 | 0.349 |
| T stage |  |  |  |
| T1-2 | Ref |  |  |
| T3-4 | 1.143 | 0.702-1.861 | 0.591 |
| N stage |  |  |  |
| N0 | Ref |  |  |
| N1 | 1.061 | 0.670-1.679 | 0.801 |
| **Metastatic site** |  |  |  |
| *Bone* |  |  |  |
| No | Ref |  |  |
| Yes | 1.465 | 0.904-2.375 | 0.121 |
| *Lung* |  |  |  |
| No | Ref |  |  |
| Yes | 1.110 | 0.699-1.764 | 0.659 |
| *Liver* |  |  |  |
| No | Ref |  |  |
| Yes | 1.197 | 0.677-2.117 | 0.536 |
| Metastatic time |  |  |  |
| Synchronous | Ref |  |  |
| Metachronous | 1.156 | 0.716-1.865 | 0.553 |
| **IMDC risk groups** |  |  |  |
| Favorable | Ref |  |  |
| Intermediate | 1.253 | 0.656-2.396 | 0.495 |
| Poor | 2.638 | 1.304-5.337 | **0.007** |
| Therapy |  |  |  |
| TKI | Ref |  |  |
| IO-TKI | 0.767 | 0.417-1.408 | 0.391 |

HR, hazard ratio; CI, confidence interval; IMDC, the International mRCC Database Consortium; TKI, tyrosine kinase inhibitor; IO-TKI, the immunotherapy-tyrosine kinase inhibitor.

The bold values indicated that p < 0.05.
