# Supplementary figures and images for: Clinical outcomes of metastatic non-clear cell renal cell carcinoma: a real-world single-centre experience
Source: Ann Med. 2026 Jan 23;58(1):2613590. doi: 10.1080/07853890.2026.2613590 (PMC12833892; doi:10.1080/07853890.2026.2613590)

Survival of rare nccmRCC pathology subtypes

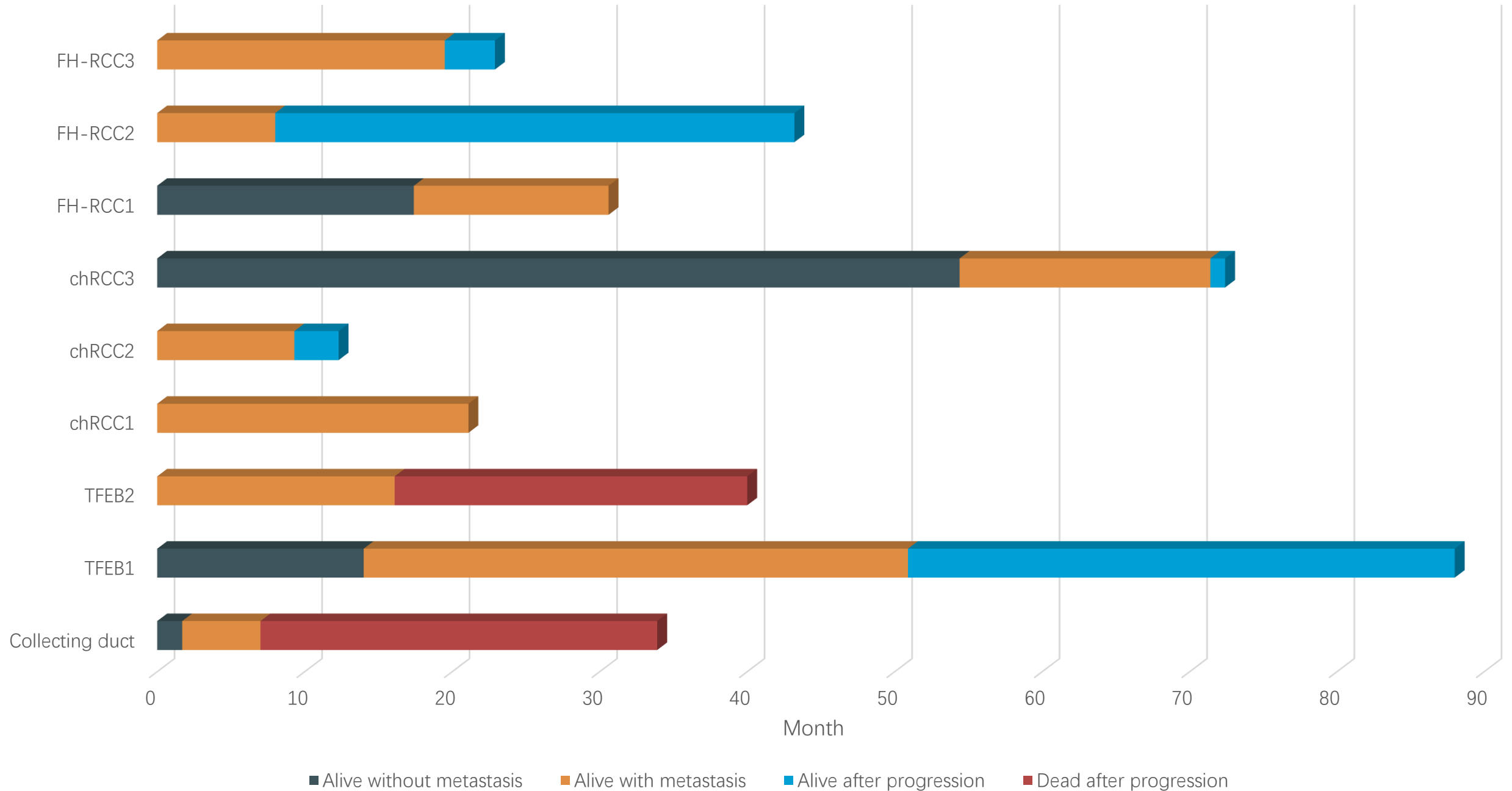

Supplement: Supplemental Material [file IANN_A_2613590_SM1202.pdf]

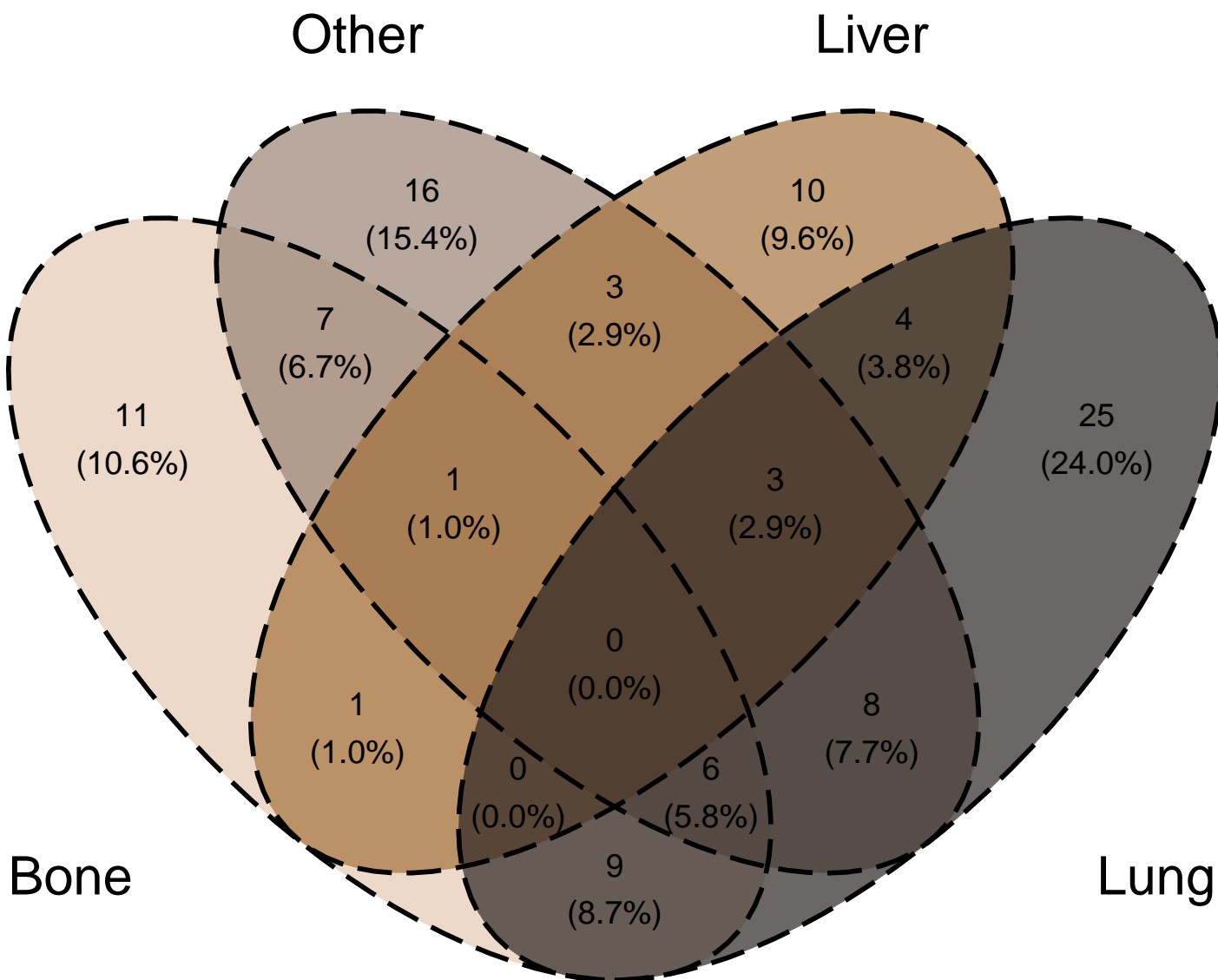

Supplement: Supplemental Material [file IANN_A_2613590_SM1197.pdf]
